# Plasma repressor element 1-silencing transcription factor levels are decreased in patients with Alzheimer's disease

**DOI:** 10.1186/s12877-022-03163-8

**Published:** 2022-06-01

**Authors:** Mingqing Wei, Jingnian Ni, Jing Shi, Ting Li, Xiaoqing Xu, Chenmeng Li, Bin Qin, Dongsheng Fan, Hengge Xie, Zhong Wang, Yongyan Wang, Tao Lu, Jinzhou Tian

**Affiliations:** 1 The Neurology Centre, Dongzhimen Hospital, Beijing University of Chinese Medicine, 100700 Beijing, China; 2 Department of Neurology, Beijing Hospital, Beijing, China; 3 Department of Neurology, Peking University Third Hospital, Beijing, China; 4 Department of Neurology, Chinese PLA General Hospital, Beijing, China; 5 Institute of Basic Research in Clinical Medicine, China Academy of Chinese Medical Sciences, Beijing, China; 6 School of Life Sciences, Beijing University of Chinese Medicine, 100029 Beijing, China

**Keywords:** Alzheimer's disease, Repressor element 1-silencing transcription, Cognition, Medial temporal lobe atrophy

## Abstract

**Background:**

Repressor element 1-silencing transcription (REST)/neuron-restrictive silencer factor is considered a new therapeutic target for neurodegenerative disorders such as Alzheimer’s disease (AD). However, the relationship between AD and REST remains unclear. This study aimed to 1) examine plasma REST levels and REST gene levels in AD patients and 2) further explore the pathological relationships between REST protein levels and cognitive decline in clinical conditions, including medial temporal lobe atrophy.

**Methods:**

Participants (*n* = 252, mean age 68.95 ± 8.78 years) were recruited in Beijing, China, and then divided into a normal cognition (NC) group (*n* = 89), an amnestic mild cognitive impairment (aMCI) group (*n* = 79), and an AD dementia group (*n* = 84) according to diagnostic criteria. All participants underwent neuropsychological assessments, laboratory tests, and neuroimaging scans (magnetic resonance imaging) at baseline. Plasma REST protein levels and the distribution of REST single nucleotide polymorphisms (SNPs) were compared among the three groups. Correlations between cognitive function, neuro-imaging results, and REST levels were determined by a multivariate linear regression analysis.

**Results:**

The plasma REST levels in both the NC group (430.30 ± 303.43)pg/ml and aMCI group (414.27 ± 263.39)pg/ml were significantly higher than that in the AD dementia group (NC vs AD dementia group, *p* = 0.034; aMCI vs AD dementia group, *p* = 0.033). There was no significant difference between the NC and aMCI groups (*p* = 0.948). No significant difference was found among the three groups regarding the genotype distribution (rs2227902 and rs3976529 SNPs) of the REST gene. The REST level was correlated with the left medial temporal lobe atrophy index (*r* = 0.306, *p* = 0.023). After 6 months of follow-up, the REST level in the NC group was positively correlated with the change in the Mini-Mental State Examination score (*r* = 0.289, *p* = 0.02).

**Conclusion:**

The plasma REST protein level is decreased in AD dementia patients, which is associated with memory impairment and left temporal lobe atrophy and may have potential value for clinical diagnosis of AD dementia.

## Background

Alzheimer's disease (AD) is a progressive neurodegenerative disease that ranges from preclinical and mild cognitive impairment (MCI) to symptoms of dementia and accounts for 60%–80% of all causes of dementia in the population older than 65 years [1]. AD-related pathological changes in the brain have been verified to include accumulation of amyloid-beta protein and neurofibrillary tangles containing hyperphosphorylated tau. However, the pathological mechanisms of AD are still unclear. Repressor element 1-silencing transcription/neuron-restrictive silencer factor (REST/NRSF) has been reported to be absent in brain tissues of patients with AD and MCI [2]. A similar trend was noted for the plasma neuronal exosomal levels [3, 4]. Hence, REST is considered a new therapeutic target for neurodegenerative disorders [5]. The neuroprotective function of REST is correlated with down regulation of genes that lead to cell death and AD pathology, and REST protects neurons from oxidative stress and amyloid-beta protein toxicity [2]. Moreover, REST and its target genes have been implicated in the pathogenesis of a number of neurodegenerative diseases. Studies have shown that the longitudinal assessment of psychomotor speed is also associated with the REST genotype (rs3796529), and statistically significant associations exist between genotypic variation and memory function at baseline (NRSF rs2227902) and in longitudinal analysis (REST rs2227902) [6]. Here, we examined REST gene and plasma protein levels to further examine REST expression. The main purpose of this study was to investigate whether REST protein and gene levels differ between participants with normal cognition and those with AD and whether the REST protein level is correlated with cognitive decline and medial temporal lobe atrophy.

## Methods

### Participants

Chinese-speaking participants aged from 50 to 85 years were recruited for this study from September 2015 to July 2019 in the memory clinic of Dongzhimen Hospital, Beijing University of Chinese Medicine. After providing informed consent, the participants underwent a standard neuropsychological assessment, laboratory tests, and neuroimaging scans. The neuropsychological assessment included examination of global cognition (Mini-Mental State Examination, MMSE) and single cognitive domains including the following [7]: (1) episodic memory (Chinese version of the Immediate and Delayed Story Recall Test, ISR and DSR) [8], (2) visual-spatial skill (Clock Drawing Test, CDT) [9], (3) executive function (Chinese version of the Trail Making Test A, TMT-A) [10], (4) language function (Chinese version of the Boston Name Test, BNT) [11], and activities of daily living (ADL) [12]. The Clinical Dementia Rating (CDR) scale was administered to assess the loss of function caused by cognitive impairment [13].

All participants were classified into the normal control (NC) group, amnestic mild cognitive impairment (aMCI) group, or AD dementia group, according to diagnostic criteria. The NC group was classified according to the Mayo Clinic criteria for healthy controls as follows [14]: participants (1) had no active neurological or psychiatric disease; (2) were not taking any psychotropic medication; (3) had no medical disorders and were not undergoing treatments that could compromise cognitive function; and (4) had normal cognitive function as determined by an MMSE score greater than 26, an ADL score less than 16, and a CDR score of 0.

Participants meeting the diagnostic criteria of the MCI Working Group of the European Consortium on Alzheimer’s Disease were included in the aMCI group according to the following [15]: (1) memory complaints reported by participants or their family members; (2) objective memory impairment (DSR score < 12.5 adjusted for age); (3) normal general cognitive function and no or minimal impairment in ADL, an MMSE score greater than 24, an ADL score less than 16, a CDR score of 0.5, and a memory domain score of 0.5 or 1; and (4) absence of dementia as determined by a clinician with experience in dementia research.

The core clinical criteria of the National Institute on Aging-Alzheimer’s Association Workgroup were used to determine probable AD [16], and an operational diagnostic standard for AD dementia was adopted based on the Chinese context as follows [17]: (1) gradual and progressive cognitive function decline over 6 months; (2) significant episodic memory impairment (DSR score < 12.5) [8] and impairment of at least one other cognitive domain (TMT-A score > 98 s, BNT-30 score ≤ 22, or CDT score ≤ 3) [10, 11]; (3) global cognitive decline evaluated by the MMSE after adjustment for education (scores of ≤ 22 for illiterate participants, ≤ 23 for participants with a primary school education, ≤ 24 for participants with a middle school education, or ≤ 26 for participants with higher education) [18–20]; (4) impaired ADL (ADL score ≥ 16) [12]; and (5) age-adjusted medial temporal lobe atrophy (MTA scale) based on coronal magnetic resonance imaging (MRI) of the brain (scores of ≥ 1.0 for participants aged ≤ 65 years, ≥ 1.5 for participants aged 66–75 years, and ≥ 2.0 for participants aged > 75 years) [21].

### MRI visual rating scale

A standard MRI scan (3.0 Tesla scanner, Siemens, Magnetom Verio, Germany) for dementia was performed on participants at the Department of Radiology, Dongzhimen Hospital, Beijing University of Chinese Medicine. The image analysis and rating procedures have been previously described in detail [21].

The MTA scale was used to assess the medial temporal lobe [22], while the global cortical atrophy scale was used to assess global cortical atrophy [23]. The posterior atrophy rating scale was used to assess posterior atrophy [24]. The medial temporal lobe atrophy index (MTAi) was used to measure the relative extent of atrophy in the medial temporal lobe in relation to global cerebral atrophy [25], which consisted of calculating the ratio of the areas of three regions that were manually traced on a single coronal MR image at the level of the inter peduncular fossa including the following: (1) the medial temporal lobe region (A); (2) the parenchyma within the medial temporal region, including the hippocampus and the para-hippocampal gyrus—the taenia fimbria and choroid plexus were excluded (B); and (3) the body of the ipsilateral lateral ventricle (C). The MTAi on both sides was determined as follows: MTAi = (A − B) × 10/C. Two clinicians who were blinded to the diagnosis and age of the participants individually analyzed the images. The result was defined as the average score from the two clinicians.

### REST plasma protein and gene levels

Before obtaining a blood sample, participants were required to fast for 8 h. After collection, blood samples were centrifuged at 3000 g for 10 min at 4 °C. Serum was separated and stored in aliquots and then kept frozen at − 80 °C until further use. All samples were centrifuged within 2 h of collection. The plasma REST level was quantified by a human-specific enzyme-linked immunosorbent assay (ELISA) kit specific for REST (Cusabio, American Research Products, Inc., Waltham, MA, USA). Blood samples were kept at room temperature for 30 min and then processed according to the product instructions. DNA was isolated from blood cells using a Blood Genomic DNA Extraction Kit (BioTeKe Corporation, Beijing, China). Polymerase chain reaction analysis was conducted for two genetic loci: rs2227902 and rs3976529. The investigator who performed the ELISA and polymerase chain reaction assays was blinded to the group allocation.

The protocol was approved by the Dongzhimen Hospital, Beijing University of Chinese Medicine Institutional Ethics Committee. The study was undertaken in accordance with the principles of the Declaration of Helsinki. Patients or their responsible caregivers provided written informed consent.

### Statistical analysis

Data analysis was performed using SPSS version 21.0 for Windows (IBM, Armonk, NY, USA). Descriptive data are presented as the mean value ± standard deviation, and categorical data are presented as counts and percentages. Group differences regarding gender, genotype, and allele distribution were compared with a Chi-square test. Because other descriptive variables including age, years of education, neuropsychological test scores, imaging scores, and REST levels were non-normally distributed, they were compared with non-parametric tests. Multivariate linear regression analysis was performed to explore the correlations between REST levels (dependent variable) and clinical features including neuropsychological and neuro-imaging variables. Statistical significance was set at a probability value of 0.05. Receiver operating characteristic (ROC) curves were produced by plotting the sensitivity against 1 − specificity of the ability of the plasma REST level to discriminate between the AD dementia and NC groups and between the aMCI and NC groups.

## Results

### Participant demographics

A total of 511 participants were screened at the memory clinic. Among these participants, 89 were classified into the NC group, 79 were classified into the aMCI group, and 84 were classified into the AD dementia group and included in this study based on the neuropsychological assessment and laboratory test results. MRI was performed in 77 participants in the NC group, 52 participants in the aMCI group, and 55 participants in the AD dementia group. The baseline characteristics and clinical information are shown in Table 1. There was no significant difference in age or gender among the three groups, and the NC and aMCI groups had a higher number of years of education than the AD dementia group.

**Table 1:** Baseline characteristics of the study participants

Items	NC (*n* = 89)	aMCI (*n* = 79)	AD dementia (*n* = 84)
Age (y)	68.60 ± 9.28	69.16 ± 8.55	69.13. ± 8.54
Male (%)	63(54.78%)	45(47.37%)	43(43.88%)
Education(y)	13.07 ± 7.25	11.04 ± 3.17*	10.83 ± 4.48*^△^
Neuropsychological tests			
n	89	79	84
MMSE	27.96 ± 1.25	26.71 ± 1.71*	17.00 ± 5.77**^△△^
ISR	25.58 ± 10.61	12.10 ± 6.40**	2.34 ± 2.75**^△△^
DSR	23.26 ± 11.73	6.03 ± 4.20**	0.60 ± 1.67**^△△^
CDT	3.95 ± 0.22	3.78 ± 0.50	2.41 ± 1.33**^△△^
TMT-A	67.74 ± 27.23	71.26 ± 29.01	122.07 ± 33.11**^△△^
CDR-SB	0.78 ± 0.98	1.42 ± 1.20*	6.31 ± 3.12**^△△^
ADL	14.08 ± 0.28	14.28 ± 0.45*	23.16 ± 6.19*△
MRI visual rating scale			
n	77	52	55
MTA-right	0.62 ± 0.76	0.98 ± 0.92*	1.76 ± 1.02**^△△^
MTA-left	0.79 ± 0.86	0.96 ± 0.91	1.89 ± 1.05**^△△^
GCA	0.74 ± 0.75	1.11 ± 0.91*	0.97 ± 0.86
PA	0.62 ± 0.66	0.94 ± 0.72**	1.12 ± 0.66**^△△^
MTAi-right	1.97 ± 1.48	2.71 ± 1.46**	3.47 ± 1.71**^△^
MTAi-left	1.71 ± 1.49	2.35 ± 1.37*	3.42 ± 1.58

*NC* normal control, *aMCI* amnestic mild cognitive impairment, *AD* Alzheimer’s disease, *MMSE* Mini Mental State Examination, *ISR* Instant Story Recall, *DSR* Delayed Story Recall, *CDT* Clock Drawing Test, *TMT-A* Trail Making Test A, *CDR-SB* Clinical Dementia Rating Sum of Boxes, *ADL* Activities of Daily Living

^*^ *p* < 0.05 for the AD dementia or aMCI group vs the NC group

^** ^*p* < 0.01 for the AD dementia or aMCI group vs the NC group

^△ ^*p* < 0.05 for the AD dementia vs the aMCI group

△△p<0.01 for AD dementia vs the aMCI group

*MRI* magnetic resonance imaging, *MTA* medial temporal lobe atrophy scale, *GCA* global cortical atrophy scale, *PA* posterior atrophy, *MTAi* medial temporal lobe atrophy index (medial temporal lobe region − the parenchyma within the medial temporal region) × 10/body of the ipsilateral lateral ventricle

### REST genotype and allele distribution

Table 2 shows the distribution of REST single nucleotide polymorphisms. No significant difference was found in the distribution of rs2227902 and rs3976529 genotypes among the three groups. We further assessed the proportion of major or minor alleles and found no significant difference among the three groups.

**Table 2:** Genotype and allele distribution of REST single nucleotide polymorphisms in study participants

·	N	Allele M/m	Genotype n(%)			p	Alleles n(%)		p
			MM	Mm	mm		M	m	
Rs2227902		G/T							
NC	89		81(92.0%)	5(5.7%)	2(2.3%)	0.772	167 (94.9%)	9(5.1%)	0.849
aMCI	79		71(89.9%)	7(8.9%)	1(1.3%)		149(94.3%)	9(5.7%)	
AD dementia	84		74(88.1%)	9(10.7%)	1(1.2%)		157(93.5%)	11(6.5%)	
Rs3976529		C/T							
NC	89		31(34.8%)	42(47.2%)	16(18.0%)	0.779	104(58.4%)	74(41.6%)	0.954
aMCI	79		26(33.3%)	42(53.8%)	12(12.8%)		94(58.8%)	66(41.2%)	
AD dementia	84		26(32.5%)	44(55.0%)	10(12.5%)		96(60.0%)	64(40.0%)	

### Correlation between REST and age

**Fig. 1:**
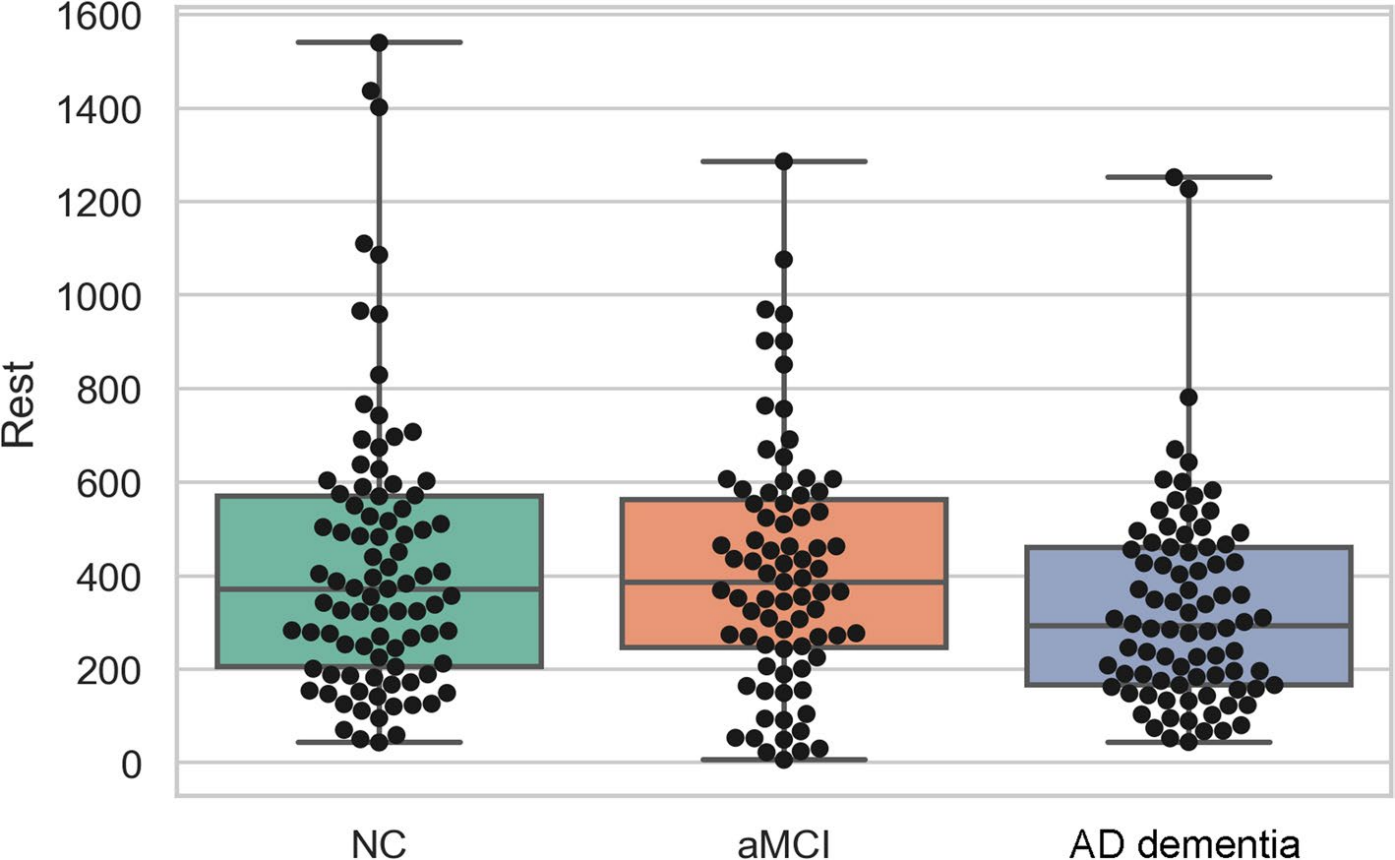
Comparison of plasma REST levels among the three groups of participants. NC, normal control; aMCI, amnestic mild cognitive impairment; AD, Alzheimer’s disease; REST, repressor element 1-silencing transcription factor; *p < 0.05 the NC or aMCI group vs the AD group

The plasma REST levels are shown in Fig. 1. A significant difference was found in the plasma REST level among the three groups (*p* = 0.048). The REST level in the NC group (430.30 ± 303.43)pg/ml was significantly higher than that in the AD dementia group (333.08 ± 222.64)pg/ml (*p* = 0.034). The plasma REST level in the aMCI group (414.27 ± 263.39)pg/ml was also significantly higher than that in the AD dementia group (*p* = 0.033). There was no significant difference between the NC and aMCI groups (*p* = 0.948).

To explore the correlation between the plasma REST level and age, we divided the NC group into three sub-groups as follows: age < 65 years, 65 ≤ age < 75 years, and age ≥ 75 years. There was no significant difference among the three age groups (*p* = 0.071). When all subjects were divided into three age sub-groups (age < 65 years, 65 ≤ age < 75 years, and age ≥ 75 years), a significant difference was found among the three sub-groups (*p* = 0.013). The REST level in the age < 65 years group (465.14 ± 284.59 pg/ml) was significantly higher than those in the other two age groups (65 ≤ age < 75 years: 371.69 ± 251.42, *p* = 0.021; age ≥ 75 years: 346.00 ± 260.82 pg/ml, *p* = 0.006).

### Correlation between the REST level and cognition

We further divided the AD dementia group into three sub-groups according to the MMSE score: mild (20 ≤ MMSE < 26), moderate (10 ≤ MMSE < 20), and severe (MMSE < 10). The results showed no significant difference in the plasma REST level among the three sub-groups with different levels of global cognitive impairment.

The correlations between the REST level and clinical features (including neuropsychological assessments and the MRI visual rating scale) were analyzed, which indicated that the left MTAi (*r* = 0.306, *p* = 0.023) and ISR (*r* = 0.526, *p* = 0.040) were both positively correlated with the REST level, while no significant correlation was found for the other variables.

### Correlation between REST level and cognitive changes after 6 months

Sixty-four participants in the NC group underwent the same neuropsychological assessments at 6 months after the first visit, and the correlation between the neuropsychological test results and the baseline REST level was calculated. The baseline REST level was correlated with the change in the MMSE score from baseline to 6 months (*r* = 0.289, *p* = 0.02). No significant correlation was found between the other neuropsychological test scores (including the DSR, ISR, CDT, TMT-A, and CDR-Sum of Boxes) and the baseline REST level.

### Sensitivity and specificity of the plasma REST level in discriminating participants with AD dementia

**Fig. 2:**
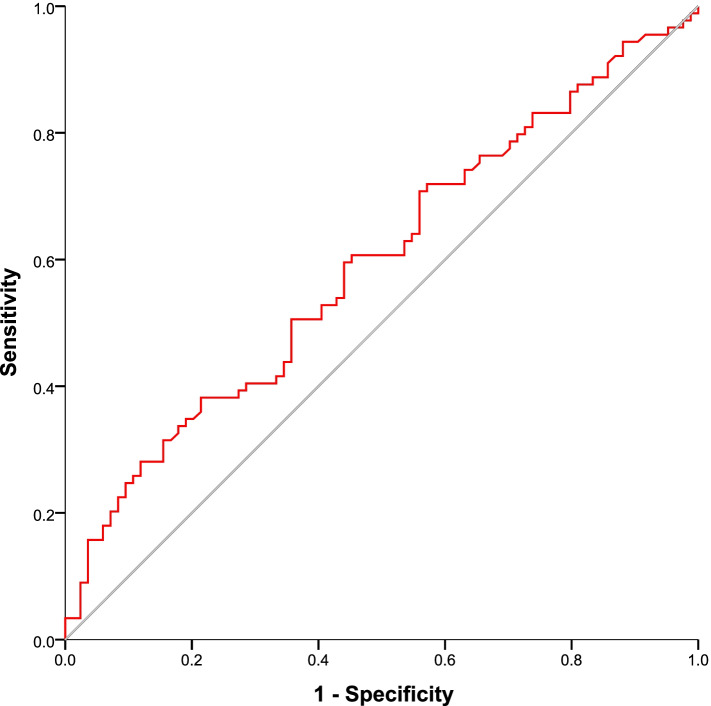
ROC curve assessing the ability of plasma REST to differentiate the NC and AD dementia groups. NC, normal control; AD, Alzheimer’s disease; REST, repressor element 1-silencing transcription factor; ROC, receiver operating characteristic

A ROC analysis was performed to calculate the cutoff score and diagnostic value of the plasma REST level for discriminating the AD dementia group from the NC group (Fig. 2). The area under the curve (AUC) was 0.593 (*p* = 0.043) (95% confidence interval [CI]: 0.509–0.678). When the cutoff value for the plasma REST level was 477 pg/ml, the sensitivity (38.2%) and specificity (78.6%) were sufficient to distinguish the AD dementia group from the NC group. The AUC for distinguishing the NC group from the aMCI group was 0.497 (95% CI: 0.409–0.585), and the AUC for distinguishing the aMCI group from the AD dementia group was 0.597 (95% CI: 0.509–0.685).

## Discussion

The results of this cross-sectional study indicated that the REST protein level in plasma was decreased in the group with mild cognitive dysfunction, while the REST expression level was markedly decreased in the AD dementia group. The REST protein level was correlated with memory function and the MTAi.

REST, as a neuro-protective factor, has received increased attention in recent years. Lu et al. first showed that REST was increasingly expressed in human cortical and hippocampal neurons during ageing. The results showed that the REST level was reduced by only 40% in individuals with MCI but was reduced by another 1.5-fold (60%) in the nucleus of cortical and hippocampal neurons in AD patients [2]. Our results were consistent with this study. In our study, the AD dementia group had a lower plasma REST level than the NC and aMCI groups. The plasma REST level was determined using an ELISA, and the trend of the plasma REST level was consistent with that of the REST level in the nucleus. The results of our study support the idea that REST might be a neuro-protective factor, and the loss of REST may lead to further development of the pathological changes associated with AD.

Studies have found that the REST protein level in neuronal nuclei of AD patients is related to the maintenance of cognitive function. Lu et al., found nearly a total absence of REST in the nuclei of prefrontal cortical neurons in the brains of AD patients, and nuclear REST levels were significantly correlated with measures of episodic, semantic, and working memory [2]. However, no studies have examined whether the plasma REST level is correlated with cognition. Our study found that the REST protein level in plasma is positively correlated with memory function, as measured by an instant memory recall test, but there was no correlation between global cognition and REST. Meanwhile, the baseline plasma REST level was correlated with the change in the MMSE score after 6 months of follow-up, indicating that the baseline REST level may predict changes in global cognition.

In addition, our study also found a positive correlation between plasma REST levels and the MTAi-left score, which was consistent with a previous study [26], suggesting that REST may serve as an independent risk marker for AD.

The notion that REST gene polymorphisms have a protective effect on patients with MCI or AD dementia remains controversial. Some studies have sequenced the rs3796529 and rs2227902 variants in European and American populations and shown that MCI patients carrying the T allele at rs3796529 have a larger hippocampal volume and slower atrophy rate. However, whether the rs3796529 variant can protect the hippocampus of people with normal cognitive function has not been shown [27]. The rs3796529 variant has been reported to have a neuro-protective effect in healthy people and MCI patients [28], but another study showed that the rs3796529 variant is not related to hippocampal volume. In contrast, the minor T allele of the rs2227902 variant seems to be related to a decreased right hippocampal volume [29], resulting in inconsistent conclusions of these studies. In the present study, no significant difference in REST gene polymorphisms was found among the three groups, and no correlation with the hippocampal volume was observed. Further studies and discussions are still needed in this area.

In current clinical practice, the plasma REST level shows relatively low sensitivity for distinguishing AD dementia patients from NC subjects and MCI patients. However, another study showed high sensitivity for distinguishing NC subjects from AD dementia patients based on the plasma REST level (CI: 100%–100%) [30]. The possible reason for this difference may be that the test method that we used was not sensitive.

### Limitations

This study has some limitations. First, the sample size was relatively small, and the above conclusion may need to be further verified with a larger sample. Second, it is unknown whether the peripheral plasma REST protein level represents the level in neurons. However, this study provides a method and basis for carrying out REST testing in clinical research.

## Conclusion

The plasma REST protein level is decreased in AD dementia patients and may be associated with memory function and left temporal lobe atrophy. Therefore, the plasma REST level may have potential value for clinical diagnosis of AD dementia. However, because of the relatively small sample size, this conclusion needs to be further verified in a larger population.

## Data Availability

The data that support the findings of this study are available on request from the corresponding authors. The data are not publicly available because of privacy or ethical restrictions.
